# A machine learning model for prediction of cardiac arrest-associated acute kidney injury in the ICU: an internal and external validation study

**DOI:** 10.3389/fmed.2025.1717973

**Published:** 2026-01-20

**Authors:** Wenbo Xu, Shenchi Cheng, Chenxi Wu, Chen Li, Tianhao Ni, Peifeng Ni, Gensheng Zhang, Mengyuan Diao, Wei Hu

**Affiliations:** 1 The Fourth School of Clinical Medicine, Zhejiang Chinese Medical University, Hangzhou, Zhejiang, China; 2 Department of Critical Care Medicine, Affiliated Hangzhou First People’s Hospital, School of Medicine, Westlake University, Hangzhou, Zhejiang, China; 3 Department of Critical Care Medicine, Zhejiang University School of Medicine, Hangzhou, Zhejiang, China; 4 Department of Critical Care Medicine, Second Affiliated Hospital, Zhejiang University School of Medicine, Hangzhou, China

**Keywords:** acute kidney injury, cardiac arrest, machine learning, MIMIC-IV database, predictive modeling

## Abstract

**Background:**

Cardiac arrest-associated acute kidney injury is common after cardiac arrest and adversely affects patient survival and disease outcomes. Early prediction of acute kidney injury is essential for guiding clinical management, especially in cardiac arrest patients admitted to the intensive care unit. Early detection of acute kidney injury can improve long-term outcomes.

**Methods:**

Data were obtained from two local hospitals and the Medical Information Mart for Intensive Care (MIMIC)-IV database. Feature selection was performed using least absolute shrinkage and selection operator regression. Model performance was evaluated using decision curve analysis and calibration curves, and the best-performing model was interpreted with SHapley Additive exPlanations.

**Results:**

This study included 873 patients from local hospitals and 719 patients from the MIMIC-IV database as an external validation cohort, least absolute shrinkage and selection operator regression identified 10 predictor variables. The logistic regression model demonstrated the best performance in predicting cardiac arrest-associated acute kidney injury, with an area under the curve of 0.958 (95% CI: 0.942–0.974) in the training set, 0.953 (95% CI: 0.920–0.987) in the internal validation set, and 0.825 (95% CI: 0.791–0.859) in the external validation set. The model was further interpreted using the SHAP framework.

**Conclusion:**

An externally validated logistic regression model incorporating 10 variables effectively predicted early acute kidney injury onset in cardiac arrest patients. The SHapley Additive exPlanations algorithm facilitated model interpretation, helping clinicians understand the contribution of each variable to acute kidney injury risk, to determine which factors contribute most significantly to patient risk.

## 1 Introduction

Cardiac arrest is an acute condition with an extremely high mortality risk. In-hospital survival rates are approximately 25%, while out-of-hospital survival can be as low as 10–12% (1). Survivors often develop post-cardiac arrest syndrome, a clinical state characterized by neurological dysfunction, systemic inflammation, and vasoregulatory failure following the return of spontaneous circulation (ROSC). This syndrome results from systemic ischemia during circulatory arrest and subsequent reperfusion injury, with acute kidney injury (AKI) being a frequent complication (2–5).

Respiratory failure, neurological dysfunction, liver failure, and acute kidney injury are common complications following cardiac arrest. Among these, cardiac arrest-associated acute kidney injury (CA-AKI) significantly impacts patient prognosis, characterized by rapid deterioration of renal function, with an incidence ranging from 37 to 80% (6). It is associated with poorer clinical outcomes, including decreased survival (7, 8) and increased intensive care unit (ICU) mortality (9). Early prediction and tailored management of AKI are therefore essential in post-arrest care. Current diagnosis relies on the Kidney Disease: Improving Global Outcomes (KDIGO) criteria, which use serum creatinine levels or urine output. However, these markers have limitations: urine output can be influenced by hemodynamic status and treatments, while rises in serum creatinine are often delayed (10). These shortcomings underscore the need for more reliable predictive tools to identify AKI earlier in this high-risk population. The development of current prediction tools is often confined to a single data center, making it difficult to generalize across diverse patient populations. Therefore, the development and application of higher-quality models, those based on multicenter data, incorporating larger patient cohorts, and validated externally are crucial for addressing the current challenges.

The integration of machine learning (ML) with electronic health records has advanced predictive capabilities across various medical fields (11–13). Yet, its application for predicting AKI following cardiac arrest remains limited. Most existing models are derived from single-center, small-scale datasets and lack external validation, which restricts their generalizability and clinical applicability. To address this gap, our study developed an ML model using multicenter data and performed rigorous internal and external validation. Our aim was to create a robust, clinically generalizable tool for early prediction of AKI in patients after cardiac arrest.

The main contributions of this study are as follows:

- A high-performance prediction model based on logistic regression was developed: We integrated multicenter clinical data and utilized LASSO regression to select 10 key features, constructing a simplified and interpretable logistic regression (LR) model for predicting AKI within 48 h after cardiac arrest. This model demonstrated exceptional discriminatory ability (AUC > 0.95) in both the training set and internal validation set.
- Rigorous external validation was conducted: We externally validated the model using the publicly available international MIMIC-IV database as an independent validation cohort. Results demonstrated robust predictive performance (AUC = 0.825) even across different patient populations and clinical settings, confirming its excellent generalization capability and potential universality.
- Provided deep clinical interpretability analysis based on SHAP: We applied the SHAP algorithm to interpret the model, not only quantifying the global importance of each predictor but also revealing the nonlinear relationship between key variables (such as SOFA score and lactate) and AKI risk, along with specific clinical risk thresholds. This transformed model outputs into understandable clinical insights.

## 2 Related works

Research on the early prediction of acute kidney injury (AKI) has progressively shifted from reliance on clinical scoring systems and novel biomarkers toward data-driven analytical models (14–16). Despite these advances, there remains a notable lack of predictive tools specifically for AKI following cardiac arrest that possess robust generalization capability. The application of ML to electronic health records (EHRs) represents a paradigm shift, allowing for the capture of complex, nonlinear relationships and offering superior predictive performance in routine intensive care settings. Notably, models developed using large ICU databases such as MIMIC have demonstrated the ability to identify AKI hours before clinical diagnosis (17).

Over the past 5 years, the deep integration of ML with EHRs has substantially improved the accuracy and real-time processing of critical illness prognostic models. For example, one study (18) utilized an improved particle swarm optimization-based gradient optimizer to tune long short-term memory networks, achieving 89.9% accuracy on gait time series data from 73 patients with Parkinson’s disease. This illustrates the potential of combining meta-heuristic algorithms with recurrent neural networks for analyzing small-sample medical time-series data. Bacanin et al. (19) proposed an extreme learning machine (ELM) optimized by the group search firefly algorithm, which outperformed nine classical meta-heuristic algorithms across 16 medical benchmark datasets, including fetal heart rate curves. This suggests ELM as a candidate for ultra-low-latency scenarios in critical care. Zivkovic et al. (20) applied an improved arithmetic optimization algorithm to a convolutional neural network-XGBoost hybrid model, achieving 99.4% accuracy on a dataset of 12,000 COVID-19 chest X-ray images. Their two-stage “convolutional features + tree model” framework provides a structural reference for the “LSTM + LightGBM” cascade adopted in the present study. Jovanovic et al. (21) employed a recurrent neural network tuned by the crayfish optimization algorithm on the PTB-XL database, attaining an F1-score of 0.995, which confirms the robustness of meta-heuristic parameter optimization in physiological signal analysis.

In summary, ML research in critical care has transitioned from a paradigm of “traditional statistics + small feature sets” toward one centered on “machine learning + large-scale feature data.” This evolution establishes a methodological foundation for our study, which focuses on predicting AKI in the specific ICU subpopulation of post-cardiac arrest patients.

## 3 Methods

### 3.1 Study population

This study included cardiac arrest patients admitted to the intensive care units (ICUs) of the First People’s Hospital of Hangzhou and the Second Affiliated Hospital of Zhejiang University between 2017 and 2024. Data from these two centers constituted the training and internal validation cohort. For external validation, we used data from the Medical Information Mart for Intensive Care IV (MIMIC-IV, version 2.2) database. Local clinical data were extracted by the investigators from the electronic medical record systems of the participating hospitals. MIMIC-IV data were retrieved using Structured Query Language (SQL) queries executed via the PostgreSQL database management system with Navicat software, focusing on patients who were admitted to the ICU following cardiac arrest.

### 3.2 Inclusion and exclusion criteria

Adult patients with cardiac arrest (defined as sustained cessation of cardiac mechanical activity requiring external cardiopulmonary resuscitation) who were admitted to the ICU for the first time, had an ICU length of stay >72 h, and were aged ≥18 years at ICU admission were included. Exclusion criteria were: (1) pregnancy; (2) pre-existing chronic kidney disease (CKD); (3) anatomical renal abnormalities (including renal transplant recipients or congenital/acquired solitary kidney).

### 3.3 Data collection

The following variables were initially collected: Demographics: Sex, age. Physical parameter: Body mass index (BMI). Medical history: Hypertension, diabetes, heart failure, myocardial infarction, cerebral infarction, chronic obstructive pulmonary disease (COPD), cirrhosis, cancer, chronic renal failure (CRF). Blood markers: Alanine aminotransferase (ALT), aspartate aminotransferase (AST), total bilirubin (TBil), blood urea nitrogen (BUN), glucose, sodium (Na), potassium (K), chloride (Cl), calcium (Ca), prothrombin time (PT), international normalized ratio (INR), initial serum creatinine (InitialCr), lactate (Lac), albumin. Blood cell counts: Hemoglobin, white blood cell count (WBC), platelet count (PLT). Interventions: Percutaneous coronary intervention (PCI), extracorporeal membrane oxygenation (ECMO), continuous renal replacement therapy (CRRT), intra-aortic balloon pump (IABP), targeted temperature management (TTM), mechanical ventilation. Blood gas analysis: Partial pressure of oxygen (PaO_2_), partial pressure of carbon dioxide (PaCO_2_), PH, bicarbonate (HCO₃^−^), base excess. Scoring systems: Charlson Comorbidity Index, Glasgow Coma Scale (GCS) score, Sequential Organ Failure Assessment (SOFA) score. Vital signs: Heart rate (HR), mean systolic blood pressure (SBP), mean diastolic blood pressure (DBP), mean arterial pressure (MAP), respiratory rate (RR), temperature. Medications: Vasoactive drugs, sodium bicarbonate, glucocorticoids, antiarrhythmic drugs. In total, 53 variables were recorded. Serum creatinine levels were also measured at 24 and 48 h after ICU admission to assess acute kidney injury according to KDIGO criteria.

### 3.4 Statistical analysis and characteristic variable screening

To ensure model consistency and generalizability across multicenter cohorts, we first aligned variables by comparing the data of the local dataset and the MIMIC-IV dataset. Only variables present in both cohorts with consistent clinical definitions were retained; variables unique to either dataset were excluded to enable external validation. For shared variables, unit conversions were applied as needed to achieve uniformity (e.g., bilirubin units were converted from mg/dL in MIMIC-IV to μmol/L to match the local dataset). Missing data were handled in two stages. First, variables with >30% missing values in either dataset were excluded. For remaining missing values, multiple imputation was performed using the mice package in R. To prevent data leakage and ensure unbiased performance evaluation, imputation was conducted separately on the training set (local data) and the external test set (MIMIC-IV data). Specifically, the imputation model was fitted only on the training set and then applied to the test set. All analyzed variables represent the mean of clinical measurements recorded during the first 3 days after ICU admission for cardiac arrest.

Baseline data were processed and analyzed using R (version 4.2.1). Categorical variables were compared with Fisher’s exact test and reported as frequencies (percentages). Continuous variables were compared with the Mann–Whitney U test and summarized as median (interquartile range). To identify significant predictors and mitigate multicollinearity, we calculated the variance inflation factor (VIF) for each variable; variables with VIF > 10 were excluded. Continuous variables were evaluated using analysis of variance to retain only those significantly associated with the outcome; categorical variables were screened using the chi-square test. All tests were two-tailed, with statistical significance set at *p* < 0.05.

Following the above steps, the remaining variables were further screened using least absolute shrinkage and selection operator (LASSO). LASSO regression is a widely used feature-selection method that penalizes regression coefficients based on their magnitude, thereby effectively eliminating non-informative variables and enhancing model interpretability.

### 3.5 Model development and explainability

Five machine learning algorithms—logistic regression (LR), random forest (RF), support vector machine (SVM), extreme gradient boosting (XGBoost), and k-nearest neighbors (KNN)—were applied to predict early AKI following cardiac arrest. Hyperparameters for each algorithm were optimized through randomized search tuning. To reduce overfitting and evaluation bias, a 5-fold cross-validation scheme was employed during training. The local cohort was randomly split into training and internal validation sets at a 7:3 ratio. Model performance was first assessed on the internal validation set. Subsequently, the finalized model was evaluated on the independent MIMIC-IV dataset for external validation, to validate universality and reliability.

Model performance was quantified using the area under the receiver operating characteristic curve (AUC), accuracy, sensitivity, specificity, F1-score, and Cohen’s kappa. Clinical utility across different risk thresholds was evaluated using decision curve analysis (DCA) and calibration curves. To enhance interpretability, SHapley Additive exPlanations (SHAP) were applied to illustrate the contribution of each predictor to the model’s output. This approach translates complex model decisions into an intuitive feature-outcome framework, supporting clinical understanding and practical application.

## 4 Results

### 4.1 Characteristics of the study population

**Figure 1:**
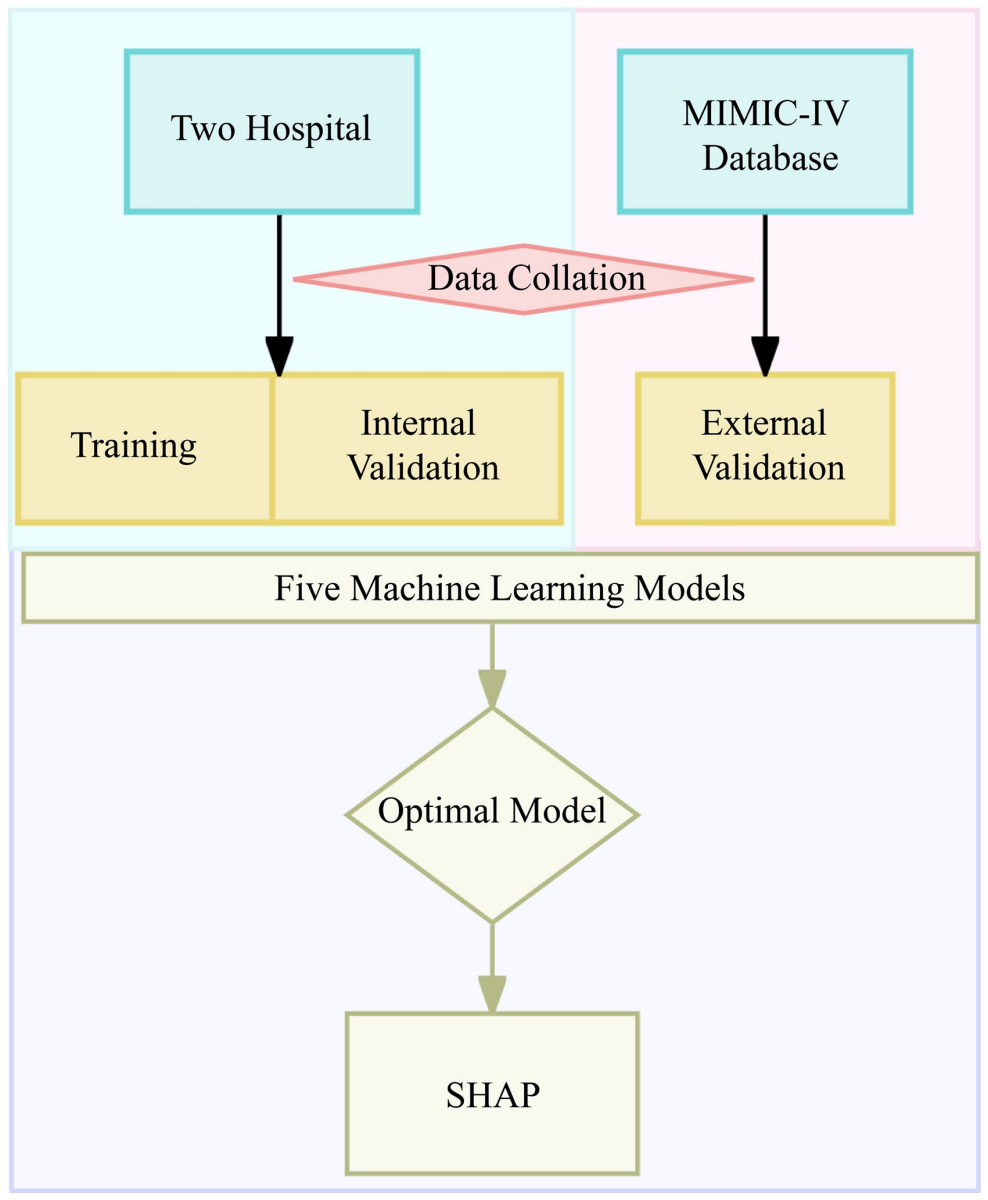
Flowchart of model development and verification.

From the two participating hospitals, 968 patients were initially identified, and 979 patients were retrieved from the MIMIC-IV database. After applying exclusion criteria to the local data, 873 patients were included, of whom 339 (39%) developed early AKI following cardiac arrest. The same inclusion and exclusion criteria were applied to the MIMIC-IV cohort, resulting in 719 patients for external validation, including 267 (37%) with early AKI. The patient selection process is summarized in Figure 1.

Baseline characteristics of the local cohort are presented in Table 1. Patients with higher BMI showed a greater incidence of early AKI, independent of hypertension, diabetes, or myocardial infarction status. Those with early AKI required more frequent use of medications (e.g., vasoactive drugs, sodium bicarbonate) and interventions such as ECMO and CRRT (Supplementary Table 1). Additionally, early-AKI patients had significantly higher HR, RR, white WBC, ALT, AST, TBil, InitialCr, BUN, glucose, K, Lac, PaCO_2_, and SOFA scores. In contrast, they exhibited lower mean SBP, temperature, hemoglobin, PLT, Cl, Ca, and GCS scores compared to non-AKI patients. Baseline characteristics of the MIMIC-IV cohort are provided in Supplementary Table 2.

**Table 1:** Baseline data table for the study population.

Variables	CA (*n* = 451)	CA-AKI (*n* = 267)	*p*-value
Age (years)	64.00 [53.00, 75.00]	65.00 [52.00, 76.50]	0.238
Gender (%)			0.033
Female	258 (57.20%)	175 (65.50%)	
Male	193 (42.80%)	92 (34.50%)	
BMI (kg/m^2^)	27.40 [23.80, 32.10]	29.30 [26.10, 33.80]	<0.001
Hypertension (%)			0.128
No	186 (41.20%)	94 (35.20%)	
Yes	265 (58.80%)	173 (64.80%)	
Diabetes (%)			0.623
No	358 (79.40%)	207 (77.50%)	
Yes	93 (20.60%)	60 (22.50%)	
Heart Failure (%)			0.315
No	310 (68.70%)	173 (64.80%)	
Yes	141 (31.30%)	94 (35.20%)	
Myocardial Infarction (%)			0.987
No	343 (76.10%)	204 (76.40%)	
Yes	108 (23.90%)	63 (23.60%)	
Cerebral Infarction (%)			0.263
No	404 (89.60%)	231 (86.50%)	
Yes	47 (10.40%)	36 (13.50%)	
COPD (%)			0.381
No	392 (86.90%)	225 (84.30%)	
Yes	59 (13.10%)	42 (15.70%)	
Cirrhosis (%)			0.030
No	442 (98.00%)	253 (94.80%)	
Yes	9 (2.00%)	14 (5.24%)	
Cancer (%)			0.626
No	402 (89.10%)	234 (87.60%)	
Yes	49 (10.90%)	33 (12.40%)	
HR (times/min)	80.00 [67.00, 92.00]	89.00 [77.00, 100.00]	<0.001
SBP (mmHg)	113.00 [105.00, 125.00]	110.00 [104.00, 119.00]	0.006
RR (times/min)	20.00 [17.00, 23.00]	22.00 [19.00, 26.00]	<0.001
Tem (°C)	37.00 [36.00, 37.00]	37.00 [36.00, 37.00]	0.319
Hemoglobin (g/L)	125.00 [106.00, 141.00]	115.00 [93.00, 136.00]	0.001
WBC (10^9^/L)	12.90 [8.95, 17.60]	13.60 [9.30, 18.20]	0.110
PLT (10^9^/L)	219.00 [170.00, 282.00]	195.00 [140.00, 263.00]	<0.001
ALT (U/L)	60.00 [28.00, 142.00]	75.00 [30.00, 252.00]	0.009
AST (U/L)	92.00 [42.50, 202.00]	119.00 [49.00, 377.00]	0.001
TBil (μmol/L)	8.55 [6.84, 13.70]	12.00 [6.84, 22.20]	<0.001
InitialCr (μmol/L)	80.00 [67.00, 92.00]	89.00 [77.00, 100.00]	<0.001
BUN (mmol/L)	18.00 [13.00, 22.00]	25.00 [17.00, 40.00]	<0.001
Glucose (mmol/L)	8.22 [6.70, 9.89]	8.89 [7.36, 12.30]	<0.001
Na (mmol/L)	139.00 [136.00, 142.00]	139.00 [136.00, 142.00]	0.979
K (mmol/L)	4.00 [3.70, 4.50]	4.40 [3.80, 5.10]	<0.001
CL (mmol/L)	104.00 [100.00, 109.00]	104.00 [99.00, 107.00]	0.075
Ca (mmol/L)	8.30 [7.70, 8.80]	8.30 [7.70, 8.90]	0.806
Lac (mmol/L)	2.70 [1.70, 4.70]	4.00 [2.00, 7.30]	<0.001
PaO_2_ (mmHg)	95.00 [54.50, 205.00]	91.00 [59.00, 206.00]	0.696
PaCO_2_ (mmHg)	45.00 [38.50, 56.00]	45.00 [36.00, 58.00]	0.381
GCS	5.00 [2.00, 9.50]	3.00 [2.00, 9.00]	0.136
SOFA	6.00 [4.00, 9.00]	10.00 [7.00, 12.00]	<0.001
CCI	4.00 [2.00, 6.00]	5.00 [3.00, 7.00]	0.001

BMI, Body Mass Index; COPD, Chronic Obstructive Pulmonary Disease; HR, Heart Rate; SBP, Systolic Blood Pressure; RR, Respiratory Rate; Tem, Temperature; WBC, White Blood Cell; PLT, Platelet; Hb, Hemoglobin; ALT, Alanine Aminotransferase; AST, Aspartate Aminotransferase; TBil, Total Bilirubin; InitialCr, Initial Creatinine; BUN, Blood Urea Nitrogen; Na, Sodium; K, Potassium; Cl, Chloride; Ca, Calcium; Lac, Lactate; PaO_2_, Partial Pressure of Oxygen; PaCO_2_, Partial Pressure of Carbon Dioxide; SOFA, Sequential Organ Failure Assessment; GCS, Glasgow Coma Scale; CCI, Charlson Comorbidity Index.

### 4.2 Feature screening

**Figure 2:**
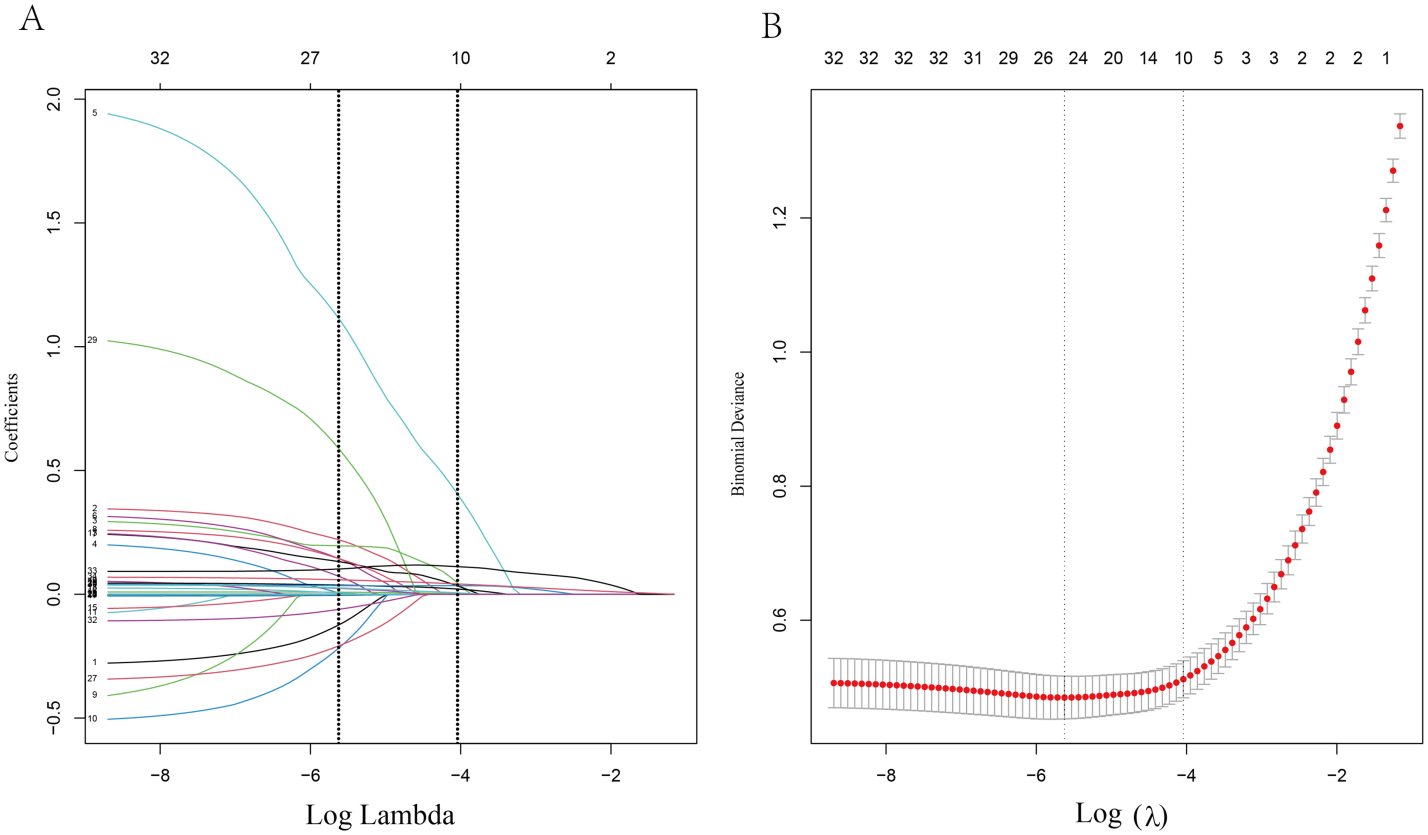
LASSO regression for feature selection in CA-AKI prediction. **(A)** Path of coefficients as a function of the regularization parameter *λ*. **(B)** Ten-fold cross-validation for optimal λ selection.

The local dataset was randomly divided into a training set (70%) and an internal validation set (30%). A total of 34 candidate variables were included in LASSO regression for feature selection. Figure 2 illustrates the trajectory of variable coefficients and the cross-validation curve of the LASSO model. The optimal penalty parameter (*λ*) that minimized the mean squared error was *λ* = 0.004, which retained 27 variables. At *λ* = 0.019 (one standard error above the minimum), 10 variables were selected: diabetes, myocardial infarction, sodium bicarbonate use, heart rate, AST, glucose, lactate, PaCO_2_, SOFA score, and initial creatinine. To balance model simplicity and predictive performance, the latter set of 10 variables was chosen for the final model.

### 4.3 Predictive modeling and performance evaluation

#### 4.3.1 Training cohort and internal validation cohort

**Figure 3:**
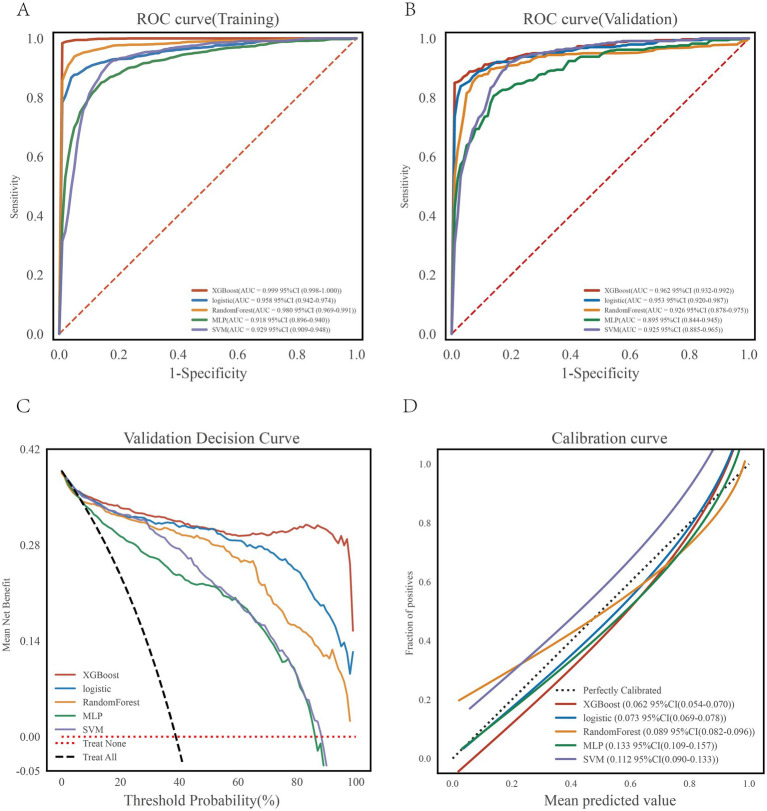
Model performance evaluation in the training set and validation sets. **(A)** Receiver operating characteristic (ROC) curve for training set. **(B)** Receiver operating characteristic (ROC) curve for validation set. **(C)** Decision curve analysis (DCA) evaluating clinical net benefit. **(D)** Calibration curve assessing agreement between predicted and observed probabilities.

Five machine learning algorithms were developed to predict early CA-AKI. In the training set, the area under the receiver operating characteristic curve (AUC) values were: XGBoost, 0.999 (95% CI: 0.998–1.000); random forest (RF), 0.980 (95% CI: 0.969–0.991); LR, 0.958 (95% CI: 0.942–0.974); support vector machine (SVM), 0.929 (95% CI: 0.909–0.948); and multilayer perceptron (MLP), 0.918 (95% CI: 0.896–0.940) (Figures 3A,B). In the internal validation set, XGBoost and LR achieved the highest accuracy. SVM showed the highest sensitivity (0.906), while LR had the highest specificity (0.949). Detailed performance metrics are presented in Tables 2, 3.

**Table 2:** Performance of the ML models in the training cohort.

Models	AUC	Accuracy	Sensitivity	Specificity	F1	Kappa
XGBoost	0.999 (0.998–1.000)	0.989	0.985	0.991	0.985	0.976
LR	0.958 (0.942–0.974)	0.925	0.872	0.958	0.900	0.840
RF	0.980 (0.969–0.991)	0.952	0.935	0.963	0.938	0.899
MLP	0.918 (0.896–0.940)	0.862	0.844	0.873	0.826	0.712
SVM	0.929 (0.909–0.948)	0.870	0.921	0.838	0.847	0.735

**Table 3:** Performance of the ML models in the internal validation cohort.

Models	AUC	Accuracy	Sensitivity	Specificity	F1	Kappa
XGBoost	0.962 (0.932–0.992)	0.915	0.882	0.936	0.890	0.821
LR	0.953 (0.920–0.987)	0.915	0.861	0.949	0.888	0.820
RF	0.926 (0.878–0.975)	0.893	0.870	0.908	0.864	0.777
MLP	0.895 (0.844–0.945)	0.836	0.817	0.848	0.796	0.660
SVM	0.925 (0.885–0.965)	0.866	0.906	0.841	0.841	0.727

Calibration curves and decision curve analysis (DCA) further compared the five models (Figures 3C,D). XGBoost and LR demonstrated the best calibration, with curves closest to the ideal diagonal. XGBoost achieved a Brier score of 0.062 and LR 0.073, both superior to the other models. DCA also indicated higher clinical net benefit for XGBoost and LR across most risk thresholds. However, while XGBoost showed near-perfect discrimination in the training set (AUC = 0.999), its performance declined in internal validation (AUC = 0.962) and more markedly in external validation (AUC = 0.812). In contrast, LR exhibited stable performance across the training (AUC = 0.958), internal validation (AUC = 0.953), and external validation (AUC = 0.825) sets, indicating better generalizability.

#### 4.3.2 External validation cohort

**Figure 4:**
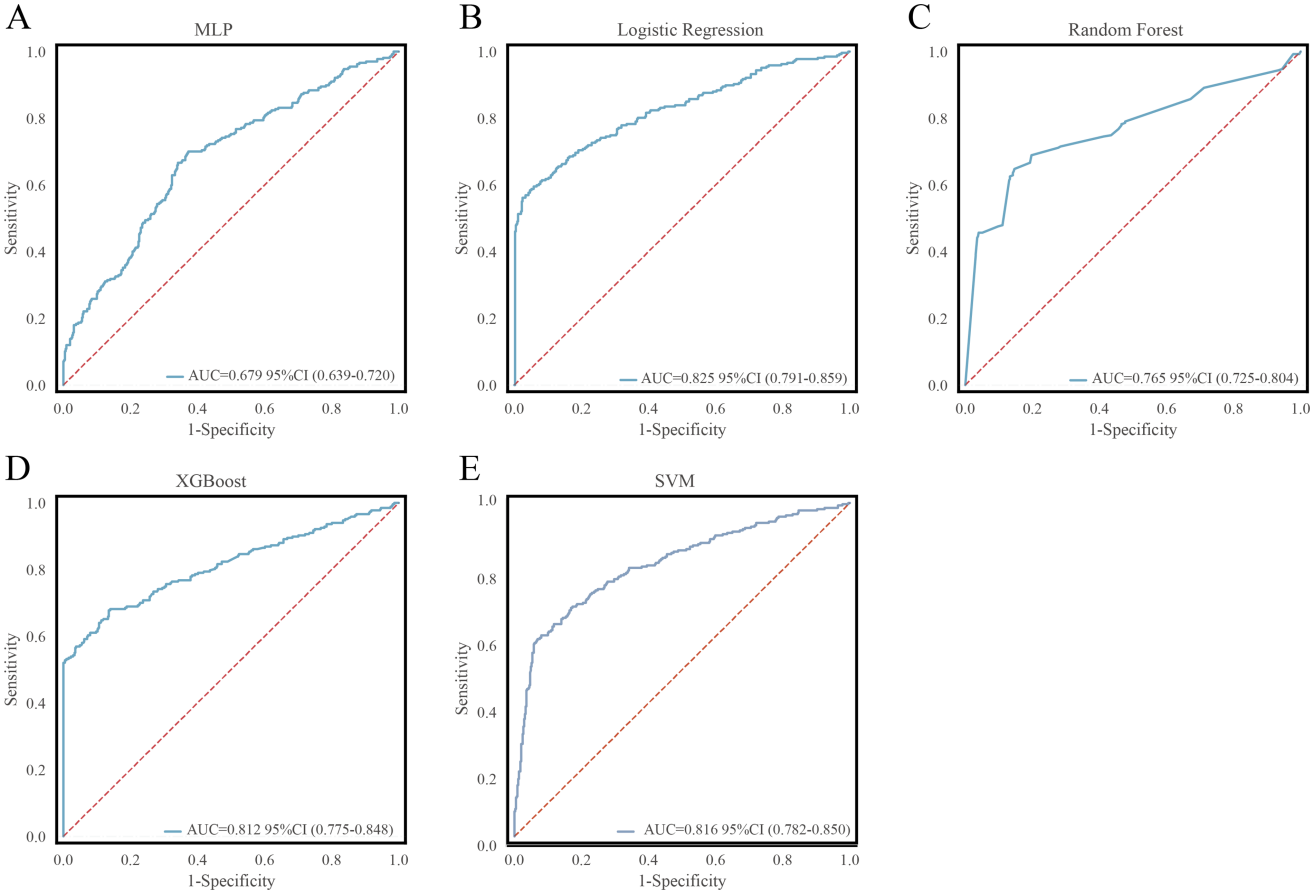
Model performance evaluation in the external validation sets. **(A)** ROC of external training for MLP; **(B)** ROC of external training for LR; **(C)** ROC of external training for RF; **(D)** ROC of external training for XGBoost; **(E)** ROC of external training for SVM.

External validation was performed on 719 patients from the MIMIC-IV database. Among the five models, LR achieved the highest AUC of 0.825 (95% CI: 0.791–0.859) (Figure 4), demonstrating robust performance despite differences in data sources. Detailed external validation metrics are listed in Table 4.

**Table 4:** Performance of the ML models in the external validation cohort.

Models	AUC	Accuracy	Sensitivity	Specificity	F1	Kappa
XGBoost	0.812 (0.775–0.848)	0.813	0.558	0.965	0.690	0.567
LR	0.825 (0.791–0.859)	0.780	0.678	0.84	0.696	0.524
RF	0.765 (0.725–0.804)	0.776	0.637	0.858	0.679	0.508
MLP	0.679 (0.639–0.720)	0.666	0.116	0.991	0.205	0.130
SVM	0.816 (0.782–0.850)	0.795	0.603	0.909	0.687	0.539

### 4.4 Interpretability analysis

**Figure 5:**
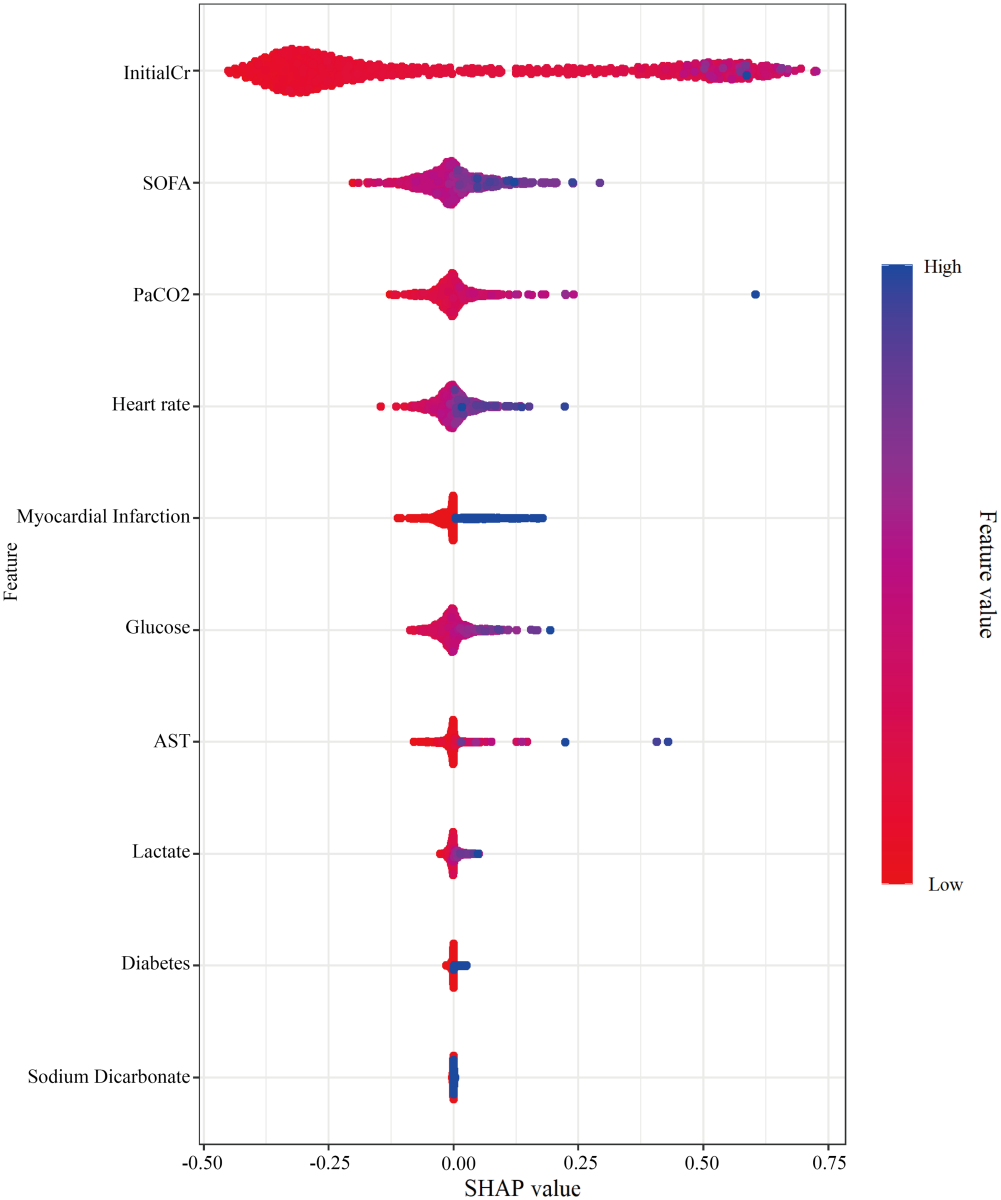
Model interpretation of the weighted ensemble model using SHAP. SHAP summary dot plot, showing the global importance, direction, and distribution of features. From top to bottom, InitialCr, SOFA, PaCO2, Heart rate, Myocardial infarction, Glucose, AST, Lactate, Diabetes, and Sodium bicarbonate.

**Figure 6:**
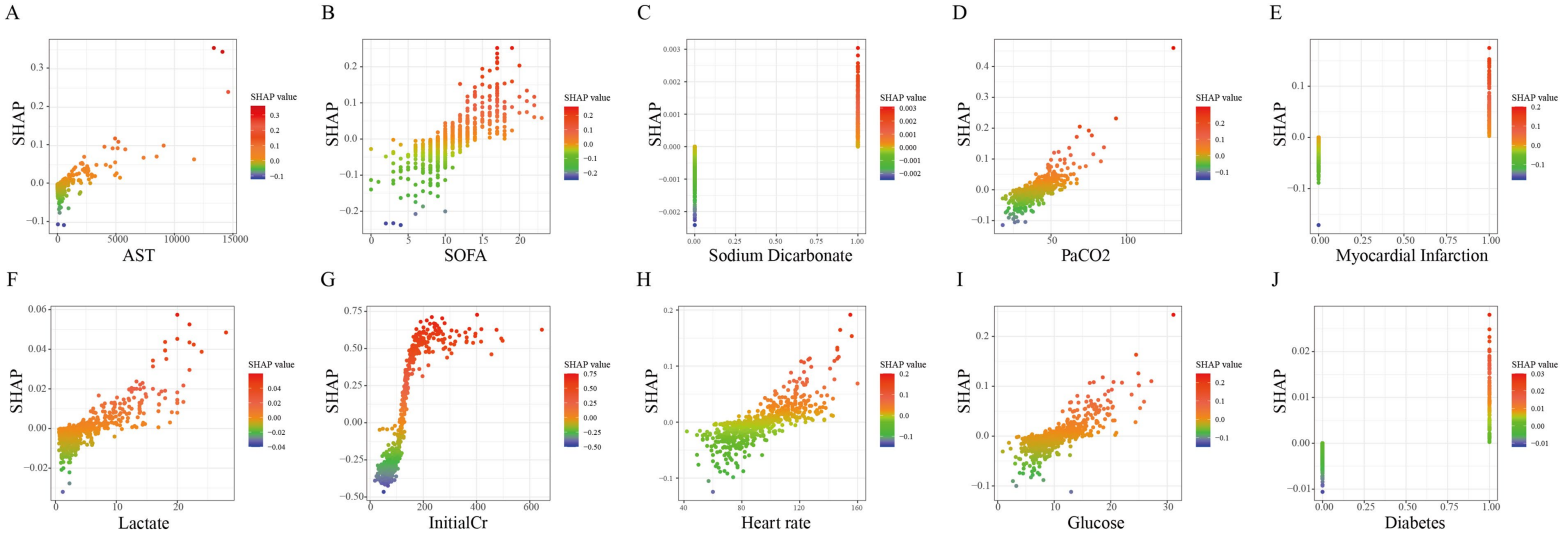
SHAP dependence plots for clinical features in the ensemble model. **(A)** AST; **(B)** SOFA; **(C)** Sodium bicarbonate; **(D)** PaCO2; **(E)** Myocardial infarction; **(F)** Lactate; **(G)** InitialCr; **(H)** Heart rate; **(I)** Glucose; **(J)** Diabetes.

The contribution of each variable in the LR model was assessed using SHapley Additive exPlanations (SHAP). Variables ranked by overall impact were: initial creatinine, SOFA score, PaCO_2_, Heart rate, myocardial infarction, glucose, AST, lactate, diabetes, and sodium bicarbonate use (Figure 5). Local interpretability analysis illustrated how specific values of each variable influenced the prediction (Figure 6), where the x-axis represents the variable value and the y-axis its corresponding SHAP value.

## 5 Discussion

In this study, we developed a predictive model for the early occurrence (within 48 h) of AKI in patients admitted to the ICU following cardiac arrest. Clinical data were collected from two hospitals between 2017 and 2024. Feature selection was performed using LASSO regression, which identified 10 predictive variables. Five machine learning algorithms were then trained and evaluated. All models performed well in the training and internal validation cohorts. However, a marked disparity in generalizability emerged during external validation using the independent MIMIC-IV database. While the LR, XGBoost, and SVM models all maintained AUC values above 0.8, the performance of the more complex models, particularly XGBoost, exhibited a significant decline from near-perfect training performance (AUC = 0.999) to a lower external validation AUC (0.812), a pattern suggestive of overfitting to the nuances of the training data. This likely stems from the model’s high complexity and capacity to capture not only the generalizable signal but also dataset-specific noise and subtle, non-generalizable patterns present in the training data. Factors such as differences in patient populations, ICU practices, and data collection protocols between the development data and external validation databases may have contributed to this decline, highlighting the challenge of achieving model portability across institutions. In contrast, the LR model demonstrated not only the highest external validation performance (AUC = 0.825) but also the most stable generalizability across all three datasets (training, internal, and external validation), and was therefore selected as the final model.

LR is a widely used multivariate method for analyzing the relationship between multiple predictors and a categorical outcome (22). In machine learning studies, LR has frequently outperformed more complex models, particularly in AKI prediction, where it has shown optimal performance in approximately 25.6% of published models—substantially higher than the 18–19% reported for other algorithms (22, 23). This advantage is often attributed to its lower propensity for overfitting, especially with limited clinical sample sizes, leading to more reliable performance in new patient populations—a finding strongly corroborated by our results. Our findings further support the robust predictive capability and superior generalizability of LR in clinical settings.

To enhance interpretability, we applied SHAP, a well-established post-hoc analysis framework that enables both global and local interpretation of model predictions (24, 25). Our analysis included global feature importance ranking and local explanation plots, the latter illustrating how individual variable values influence the prediction for specific patients.

During cardiac arrest, systemic ischemia–reperfusion injury induces oxidative stress, inflammatory mediator release, mitochondrial dysfunction, and microcirculatory impairment in renal tissue. Reduced oxidative phosphorylation exacerbates reactive oxygen species production, contributing to kidney damage, the accumulation of reactive oxygen species leads to apoptosis and kidney damage (26, 27). Clinical studies have consistently linked post-cardiac arrest AKI with decreased survival (28, 29), underscoring the importance of early detection and intervention for improving outcomes.

Previous attempts to predict AKI after cardiac arrest have been limited. Lin et al. used LASSO and LR to select four predictors from 15 clinical indicators (12). Hou et al. developed a model based on shock status, CRP, LDH, and ALP, achieving an AUC of 0.731 using the Dryad database (30). However, these models were derived from single-center data, lacked external validation, and did not incorporate a comprehensive set of clinically relevant variables, potentially omitting important predictors. In contrast, our study utilized multicenter data for model development, performed internal validation, and conducted external validation using MIMIC-IV. By initially evaluating 53 clinical indicators, we aimed to improve the comprehensiveness and robustness of the predictive model.

Our analysis identified several admission factors associated with early AKI in post-cardiac arrest patients: InitialCr, SOFA score, HR, AST, glucose, Lac, and PaCO_2_. Additionally, diabetes, history of myocardial infarction, and the need for sodium bicarbonate were linked to increased AKI risk.

InitialCr reflects baseline renal function at ICU admission and served as a strong predictor in our model. The SHAP analysis indicated a distinct increase in AKI risk when InitialCr exceeded 150 μmol/L, providing a practical threshold that may aid clinicians in risk stratification when prior renal function data are unavailable. SOFA score, a well-established measure of multi-organ dysfunction, was also significantly associated with AKI risk. SHAP based local interpretation identified a SOFA score >10 as a critical risk threshold, beyond which the predicted probability of AKI rose markedly. Elevated blood glucose-particularly levels >10 mmol/L and especially >20 mmol/L was associated with higher SHAP values and increased AKI risk. This observation aligns with prior evidence suggesting that intensive glycemic control can reduce AKI incidence in critically ill patients (31, 32). HR > 100 beats per minute emerged as another relevant predictor, consistent with findings from earlier predictive models (12, 33–35). Elevated HR may reflect underlying physiological stress such as infection, hypovolemia, or cardiac dysfunction, each of which can predispose patients to AKI. Hypercapnia (PaCO_2_ > 50 mmHg) was associated with elevated AKI risk, likely mediated through reduced renal blood flow, as suggested in previous studies (36–38). Similarly, lactate levels >10 mmol/L were strongly predictive, underscoring the role of tissue hypoperfusion and acidosis in AKI pathogenesis-a context frequently accompanying the use of sodium bicarbonate (39, 40). Aspartate aminotransferase (AST), a marker of hepatic and muscular injury, also contributed to model predictions. Its inclusion aligns with prior studies where AST served as a predictor in trauma-and burn-associated AKI (41, 42), suggesting that systemic injury and inflammation may further exacerbate renal vulnerability after cardiac arrest.

Collectively, these predictors highlight the multifactorial nature of CA-AKI, incorporating elements of baseline renal reserve, systemic organ dysfunction, metabolic stress, and perfusion-related injury. This suggests that patients with baseline creatinine levels above this threshold should initiate enhanced monitoring and renal protection strategies, even if they do not meet acute kidney injury (AKI) criteria. A rapid increase in SOFA score or a score >10 should be regarded as a warning signal, prompting prioritized assessment of hemodynamics and optimization of systemic supportive therapy. Concurrently, intensified glycemic control (target <10 mmol/L), management of heart rate (HR > 100 bpm), investigation of reversible causes, and optimization of volume and cardiac output serve as feasible interventions for AKI prevention. In mechanically ventilated patients, avoid prolonged permissive hypercapnia by adjusting respiratory parameters, particularly in those at risk for AKI. Persistent lactate elevation indicates the need for aggressive restoration of tissue perfusion, not merely correction of acidosis; sodium bicarbonate use requires careful evaluation. Significant AST elevation should be interpreted as a marker of multiorgan injury, not solely as an indicator of liver function.

This study has several limitations. First, differences in variable availability between the local datasets and MIMIC-IV necessitated the exclusion of some clinically relevant factors (such ascardiac arrest location and resuscitation timing), which may affect model completeness. Second, missing data and potential subjectivity in clinical assessments (e.g., GCS scores) could introduce inaccuracies. Third, although the model demonstrated good generalizability in internal and external validation, further prospective validation in independent cohorts is needed to confirm its clinical utility and strengthen the evidence for routine application.

## 6 Conclusion

In this study, we constructed a LR model based on 10 clinical variables to predict the early onset of CA-AKI. The model demonstrated good discriminatory power and generalization ability in both internal and external validation, confirming its feasibility for predicting CA-AKI. We employed the SHAP model to elucidate the contribution of each variable, enabling clinicians to gain deeper insights into disease prediction and the underlying mechanisms by which relevant clinical data influence the occurrence of CA-AKI.

## Data Availability

Publicly available datasets were analyzed in this study. This data can be found at: data were obtained from the MIMIC database. The first author, Wenbo Xu, has completed a series of mandatory training courses and obtained authorization to extract data from the database (certification number: 62628171). Due to ongoing research requirements, the local data are not currently available for release. Upon completion of subsequent studies, interested parties may contact the first author if data are required.
