# Supplementary material for: A machine learning model for prediction of cardiac arrest-associated acute kidney injury in the ICU: an internal and external validation study
Source: Front Med (Lausanne). 2026 Jan 20;12:1717973. doi: 10.3389/fmed.2025.1717973 (PMC12864462; doi:10.3389/fmed.2025.1717973)
Supplement: Supplementary file 1 [file Table_1.DOCX]

Supplementary Material

# Supplementary Figures and Tables

Supplementary Tables 1. Medical use and manipulation of patients included in the study cohort at local hospitals.

| Variables | CA (n=451) | CA-AKI (n=267) | P.value |
| --- | --- | --- | --- |
|  |  |  |  |
| Vasoactive Drugs (%) | | | <0.001 |
| No | 101 (22.4%) | 22 (8.24%) |  |
| Yes | 350 (77.6%) | 245 (91.8%) |  |
| Sodium Dicarbonate (%) | | | <0.001 |
| No | 376 (83.4%) | 130 (48.7%) |  |
| Yes | 75 (16.6%) | 137 (51.3%) |  |
| Glucocorticoid (%) | | | 0.572 |
| No | 314 (69.6%) | 192 (71.9%) |  |
| Yes | 137 (30.4%) | 75 (28.1%) |  |
| Antiarrhythmic Drugs (%) | | | 0.461 |
| No | 67 (14.9%) | 46 (17.2%) |  |
| Yes | 384 (85.1%) | 221 (82.8%) |  |
| PCI (%) | | | 0.803 |
| No | 417 (92.5%) | 249 (93.3%) |  |
| Yes | 34 (7.54%) | 18 (6.74%) |  |
| ECMO (%) | | | 0.259 |
| No | 435 (96.5%) | 252 (94.4%) |  |
| Yes | 16 (3.55%) | 15 (5.62%) |  |
| CRRT (%) | | | <0.001 |
| No | 432 (95.8%) | 219 (82.0%) |  |
| Yes | 19 (4.21%) | 48 (18.0%) |  |
| IABP (%) | | | 1 |
| No | 442 (98.0%) | 262 (98.1%) |  |
| Yes | 9 (2.00%) | 5 (1.87%) |  |
| TTM (%) | | | 0.11 |
| No | 431 (95.6%) | 262 (98.1%) |  |
| Yes | 20 (4.43%) | 5 (1.87%) |  |
| Mechanical Ventilation (%) | | | 1 |
| No | 4 (0.89%) | 2 (0.75%) |  |
| Yes | 447 (99.1%) | 265 (99.3%) |  |

Supplementary Tables 2. Baseline data for external validation cohort patients.

| Variables | CA (n=534) | CA-AKI (n=339) | P.value |
| --- | --- | --- | --- |
|  |  |  |  |
| Age (M [IQR]) | 60.0 [48.0, 70.0] | 60.0 [49.0, 73.0] | 0.184 |
| Gender (%) | | | 0.001 |
| FeMale | 193 (36.1%) | 86 (25.4%) |  |
| Male | 341 (63.9%) | 253 (74.6%) |  |
| BMI (M [IQR]) | 22.7 [20.2, 25.1] | 23.7 [21.3, 26.2] | <0.001 |
| Hypertension (%) | | | <0.001 |
| No | 338 (63.3%) | 171 (50.4%) |  |
| Yes | 196 (36.7%) | 168 (49.6%) |  |
| Diabetes (%) | | | <0.001 |
| No | 469 (87.8%) | 263 (77.6%) |  |
| Yes | 65 (12.2%) | 76 (22.4%) |  |
| Heart Failure (%) | | | 0.001 |
| No | 421 (78.8%) | 231 (68.1%) |  |
| Yes | 113 (21.2%) | 108 (31.9%) |  |
| Myocardial Infarction (%) | | | <0.001 |
| No | 460(86.1%) | 260 (76.7%) |  |
| Yes | 74(13.9%) | 79 (23.3%) |  |
| Cerebral Infarction (%) | | | 0.788 |
| No | 460 (86.1%) | 289 (85.3%) |  |
| Yes | 74 (13.9%) | 50 (14.7%) |  |
| COPD (%) | | | 0.513 |
| No | 512 (95.9%) | 321 (94.7%) |  |
| Yes | 22 (4.12%) | 18 (5.31%) |  |
| Cirrhosis (%) | | | 1 |
| No | 525 (98.3%) | 333 (98.2%) |  |
| Yes | 9 (1.69%) | 6 (1.77%) |  |
| Cancer (%) | | | 0.519 |
| No | 475 (89.0%) | 307 (90.6%) |  |
| Yes | 59 (11.0%) | 32 (9.44%) |  |
| Vasoactive Drugs (%) | | | <0.001 |
| No | 83 (15.5%) | 14 (4.13%) |  |
| Yes | 451 (84.5%) | 325 (95.9%) |  |
| Sodium Dicarbonate (%) | | | <0.001 |
| No | 371 (69.5%) | 135 (39.8%) |  |
| Yes | 163 (30.5%) | 204 (60.2%) |  |
| Glucocorticoid (%) | | | 0.051 |
| No | 336 (62.9%) | 190 (56.0%) |  |
| Yes | 198 (37.1%) | 149 (44.0%) |  |
| Antiarrhythmic Drugs (%) | | | 0.519 |
| No | 303 (56.7%) | 184 (54.3%) |  |
| Yes | 231 (43.3%) | 155 (45.7%) |  |

Supplementary Tables 2 (continued)

| Variables | CA (n=534) | CA-AKI (n=339) | P.value |
| --- | --- | --- | --- |
|  |  |  |  |
| PCI (%) | | | 0.025 |
| No | 463 (86.7%) | 274 (80.8%) |  |
| Yes | 71 (13.3%) | 65 (19.2%) |  |
| ECMO (%) | | | <0.001 |
| No | 454 (85.0%) | 248 (73.2%) |  |
| Yes | 80 (15.0%) | 91 (26.8%) |  |
| CRRT (%) | | | <0.001 |
| No | 408 (76.4%) | 120 (35.4%) |  |
| Yes | 126 (23.6%) | 219 (64.6%) |  |
| IABP (%) | | | 0.042 |
| No | 494 (92.5%) | 299 (88.2%) |  |
| Yes | 40 (7.49%) | 40 (11.8%) |  |
| TTM (%) | | | 0.015 |
| No | 200 (37.5%) | 99 (29.2%) |  |
| Yes | 334 (62.5%) | 240 (70.8%) |  |
| Mechanical Ventilation (%) | | | 0.224 |
| No | 14 (2.62%) | 4 (1.18%) |  |
| Yes | 520 (97.4%) | 335 (98.8%) |  |
| HR (M [IQR]) | 89.0 [75.2, 103] | 96.0 [81.0, 114] | <0.001 |
| SBP Mean (M [IQR]) | 123 [110, 135] | 116 [103, 128] | <0.001 |
| RR (M [IQR]) | 16.0 [15.0, 18.0] | 16.0 [15.0, 19.5] | 0.016 |
| Tem (M [IQR]) | 36.5 [35.9, 37.2] | 36.3 [35.6, 37.0] | 0.004 |
| Hemoglobin (M [IQR]) | 117 [88.0, 133] | 102 [77.0, 126] | <0.001 |
| WBC (M [IQR]) | 13.4 [9.83, 18.4] | 14.1 [9.55, 20.4] | 0.322 |
| PLT (M [IQR]) | 156 [114, 220] | 127 [74.5, 195] | <0.001 |
| ALT (M [IQR]) | 76.5 [39.2, 151] | 139 [48.5, 350] | <0.001 |
| AST (M [IQR]) | 123 [59.0, 245] | 313 [106, 740] | <0.001 |
| Total Bilirubin (M [IQR]) | 15.6 [11.2, 23.3] | 19.6 [12.6, 33.1] | <0.001 |
| InitialCr (M [IQR]) | 89.0 [75.2, 103] | 96.0 [81.0, 114] | <0.001 |
| BUN (M [IQR]) | 6.50 [5.00, 8.30] | 11.3 [8.10, 16.3] | <0.001 |
| Glucose (M [IQR]) | 9.00 [7.00, 11.7] | 9.60 [7.20, 13.0] | 0.028 |
| Na (M [IQR]) | 144 [141, 148] | 146 [142, 152] | <0.001 |
| K (M [IQR]) | 3.85 [3.52, 4.25] | 4.00 [3.59, 4.56] | 0.001 |
| CL (M [IQR]) | 108 [105, 112] | 107 [103, 112] | 0.001 |
| Ca (M [IQR]) | 2.09 [1.99, 2.19] | 2.07 [1.93, 2.18] | 0.04 |
| Lac (M [IQR]) | 2.40 [1.50, 4.57] | 4.80 [2.30, 10.7] | <0.001 |
| PaO2 (M [IQR]) | 129 [98.3, 165] | 129 [100, 168] | 0.67 |
| PaCO2 (M [IQR]) | 38.0 [33.0, 44.0] | 38.0 [32.0, 46.5] | 0.442 |
| GCS (M [IQR]) | 4.00 [3.00, 5.00] | 3.00 [3.00, 4.00] | <0.001 |
| SOFA (M [IQR]) | 9.00 [8.00, 11.0] | 13.0 [11.0, 16.0] | <0.001 |
| Charlson.s Index (M [IQR]) | 3.00 [2.00, 5.00] | 4.00 [2.00, 5.00] | 0.042 |
